# Females of HbAS genotype have reduced concentration of the malaria protective *deoxyhemoglobin S* than males

**DOI:** 10.1371/journal.pone.0203455

**Published:** 2018-09-11

**Authors:** John N. Waitumbi, Carolyne M. Kifude, Carol W. Hunja, Bernhards R. Ogutu

**Affiliations:** 1 Walter Reed Project, Kenya Medical Research Institute, Kisumu, Kenya; 2 South Eastern Kenya University, School of Pure and Applied Sciences, Department of Biology, Kitui, Kenya; Pennsylvania State University College of Medicine, UNITED STATES

## Abstract

The quantity of the intra-erythrocytic *deoxyhemoglobin S* (Hb S) affects the level of protection against malaria and also the sickling phenomenon. This study reports on significantly lower concentration of Hb S in females than males. Data came from 350 children, aged 12–47 months who participated in a phase 2b malaria vaccine trial. Hemoglobinopathy and G6PD deficiency typing was necessary to ascertain equal representation of these malaria protective traits across the vaccine cohorts. Hemoglobin types (HbAA, HbAS) and % Hb S were evaluated by HPLC. Alpha thalassemia (alpha-thal) and G6PD genotypes were evaluated by PCR. The overall prevalence for HbAS was 20%, 46% for 3 alpha genes and 10% for 2 alpha genes and 14% for G6PD A-. More females of HbAS/αα/αα genotype had low Hb S than males and had mean % Hb S of 37.5% ± 5.4 SD, compared to 42.0% ± 2.5 SD in males of same genotype (P = 0.018). Consistent with reduction of the malaria protective Hb S in females, parasite load in females was nearly twice that of males but the difference was not statistically significant. The X-chromosome linked G6PD deficiency did not influence the level of Hb S. We conclude that, the low Hb S in these females explains the resultant higher malaria parasite load. We speculate that the low Hb S in females could also explain observations suggesting that the sickling phenomenon tends to be less severe in females than males.

## Introduction

The pathogenic stage of malaria infection occurs following invasion of the red blood cell (RBC). It is therefore not surprising that all aspects of RBC, including the hemoglobin (S, C, E and thalassemia), membrane antigens (Duffy antigen, ovalocytosis and blood group O) and enzymes (G6PD) are involved in protection against malaria [1]. More than half a century ago, Haldane suggested that heterozygous traits for sickle cell and thalassemia (α^+^-thal) provide selective advantage against malaria [2] and to this day, these two RBC polymorphisms continue to evoke great interest [3–6]. Like sickle cell and *α*^+^-thal, X-chromosome linked G6PD deficiency has been shown to confer protection against malaria [7, 8], with one study showing a staggering 50% reduced risk for severe malaria [9]. Similar results have not been reproduced in many studies, probably because of incomplete correlation of phenotype and genotype especially in female heterozygotes where random inactivation of the two X chromosomes during embryological development leads to a mosaic of RBCs (G6PD normal and G6PD deficient) [2]

It is not uncommon for multiple genes that confer resistance to malaria to exist in the same individual, and in some cases, their interactions result in a phenotype that differs from what would be expected if the loci were inherited independently. This phenomenon, referred to as epistasis i.e. the interaction between two or more genes to control a single phenotype, is exemplified by co-inheritance of HbAS and α^+^-thal [10, 11] in which case, the resultant relative fitness differs from what would be expected if the loci were expressed independently [11–15]. In two of these studies [11 and 16], co-inheritance of *α*^+^-thal and sickle-cell disease (SCD) ameliorated the hematologic severity of SCD, thus acting as a genetic modifier of sickling phenomenon. In another study conducted in Kilifi [14], co-inheritance of HbAS and *α*^+^-thal was shown to lead to loss of malaria protection that would normally be afforded by each condition when inherited individually. The epistatic gene in these cases was α^+^-thal, whereby the relative deficiency in *α*^+^-thal genes in combination of HbAS or HbSS causes decreased intra-erythrocytic concentration of Hb S. The importance of the Hb S concentration in protection and or susceptibility to malaria has been corroborated by a murine study that reported low *P*. *yoelii* parasitemia in transgenic mice engineered to have high Hb S compared to severe infections and death in those with low Hb S [16].

A number of studies have reported that males with sickle cell disease experience higher morbidity and mortality than females [17–20]. Various explanations have been proposed for this gender effect, including higher fetal hemoglobin and F cell levels in females with sickle cell disease than males [21–23]. Recently, Gladwin *et al*. attributed this to increased bioavailability of nitric oxide in women than men [24]. This notwithstanding, it is well established that the pathologies associated with the sickling phenomenon are triggered by the polymerization of Hb S [25] and that, the more Hb S, the higher the pathology [16]. For the first time, this study reports on significantly lower concentration of Hb S in females compared to males which probably explains the amelioration of sickling in females, but could reduce protection against malaria for female sickle trait carriers.

## Methods

The study data came from 400 healthy subjects aged 12–47 months who were enrolled into a Phase 2 safety, immunogenicity and efficacy trial of malaria vaccine FMP1/AS02. The investigators adhered to the policies for protection of human subjects as prescribed in AR 70–25. Further details of the study site, ethical approvals, enrollment, blood draw, microscopic examinations and clinical data have been published before [26] and the trial is recorded at Clinicaltrials.gov: NCT00223990. Because hemoglobinopathies and G6PD deficiency are associated with protection against malaria [2–6, 27] typing of these traits was necessary to ascertain equal representation across the vaccine cohorts. Of the 400 subjects, 350 (165 females and 185 males) provided genotyping data used to calculate prevalence of hemoglobin types, G6PD deficiency and alpha thalassemia deletions. EDTA blood for genotyping was collected at screening. Study participants contributed multiple malaria parasitaemia data collected during sick visits that occurred throughout the 265 days follow-up period. *P*. *falciparum* parasite densities were determined by microscopy as described before [28, 29].

### Hemoglobin typing by high performance liquid chromatography

Analysis of hemoglobin type was done using the VARIANT I™ system using the β- Thalassemia Short Program (Bio-Rad Laboratories). All the HPLC reagents, buffers and test components were purchased from the manufacturer and used according to the manufacturer’s recommendations. Briefly, 5 μL of whole blood was diluted in 1000 μL of haemolysis buffer and passed through a separation cartridge. The variant machine then separates and quantifies the normal Hb A, Hb A_2_ and Hb F as well as the variant hemoglobins such as Hb S, C, D and E.

### Detection of 3.7 *α*^+^-thal deletions by PCR

DNA was isolated from EDTA blood using QIAamp DNA Blood mini Kits (QIAGEN Inc., CA). The *α*^+^-thal status of the children was determined as described before [30].

### G6PD genotyping

The different loci of the G6PD gene known to carry the common African mutations were amplified and then digested by restriction enzymes exactly as described previously [31]. Existence of a single nucleotide polymorphism (SNP) at position 376 A→G which creates a restriction site for *Fok*I was first determined and used to distinguish the G6PD*B (wild) from G6PD*A (potentially deficient). This was followed by identification of SNPs at positions 202 G→A, 680 G→T, 968 T→C which create restriction sites for *NLa*III, *Bst*NI & *NCi*I respectively. These SNPs define G6PD deficient individuals, designated G6PD*A-.

### Statistical methods

The difference in proportion of Hb S by alpha thalassemia genotypes in males and females was tested using a one way ANOVA test while parasite densities were compared using the Mann–Whitney U-test. To rule out the possibility of the influence of X-linked G6PD, we used Fishers exact test to check for any confounding association between G6PD status and gender. Chi-square tests were used to evaluate pairwise associations between the distributions of the three genotypes. A test of trend was done for parasitemia by Kruskal-Wallis. All statistical analysis were performed using Prism 5.0 (GraphPad Software, Inc., San Diego, CA) with two-tailed tests and an alpha value of <0.05 considered statistically significant.

## Results

### Prevalence of RBC polymorphisms reflect a high malaria attack rate

During the study, there were 1,024 sick visits (mean = 3, range = 1–8) of which 867 (84.7%) had malaria parasitemia. Consistent with this high malaria attack rate is a high prevalence for RBC polymorphisms (Tables 1–3): 20% for HbAS, 14% for G6PD*A- (6% hemizygous males, 7% heterozygous females, 1% homozygous females), and an even higher prevalence of 3.7 α^+^-thal deletions in heterozygous (-α/αα, 46%) and homozygous (-α/-α, 10%) forms. As shown in Table 1, HbAA assorted with –α/αα in 125 individuals (45%), –α/–α in 26 individuals (9%) and αα/αα in 129 individuals (46%), while HbAS assorted with –α/αα in 35 individuals (50%), –α/–α in 8 individuals (11%) and αα/αα in 27 individuals (39%). No significant pair-wise association was detected between any of these interaction (P = 0.50). Table 2 shows the assortment of hemoglobin genotypes with G6PD genotypes. HbAA assorted with G6PD*A in 76 individuals (28%), G6PD*A- in 33 individuals (12%) and G6PD*B in 170 individuals (61%), while HbAS assorted with G6PD*A in 22 individuals (31%), G6PD*A- in 8 individuals (11%) and G6PD*B in 41 individuals (58%). No significant pair-wise association was detected between any of these interactions (P = 0.91). Table 3 shows how α-globin gene numbers assorted with G6PD genotypes. –α/–α assorted with G6PD*B in 23 individuals (68%), G6PD*A in 9 individuals (27%) and G6PD*A- in only 2 individuals (6%). –α/αα assorted with G6PD*B in 94 individuals (59%), G6PD*A in 50 individuals (31%) and G6PD*A- in 16 individuals (10%) while αα/αα assorted with G6PD*B in 94 individuals (60%), G6PD*A in 39 individuals (25%) and G6PD*A- in 23 individuals (15%). No pair-wise association was detected between any of these associations (P = 0.39). Neither was a three-way association among the markers found (P = 0.54). No differences between genders were detected for hemoglobin genotypes (P = 0.97), α-globin gene number (P = 0.65) or G6PD genotype (P = 0.22).

**Table 1:** Prevalence and assortment of hemoglobin genotype (HbAA and HbAS) and α-globin gene numbers.

HEMOGLOBIN	ALPHA GENES			
GENOTYPE	2	3	4	TOTAL
AA	25	124	127	276
	9%	45%	46%	79%
AS	9	36	29	74
	12%	49%	39%	21%
TOTAL	34	160	156	350
	10%	46%	45%	

**Table 2:** Prevalence and assortment of hemoglobin genotype (HbAA and HbAS) and G6PD genotypes.

HEMOGLOBIN		G6PD GENOTYPE	
GENOTYPE	A	A-	E
AA	76	33	16
	28%	12%	61
AS	22	8	4
	30%	11%	55%
TOTAL	98	41	21
	28%	12%	60%

**Table 3:** Prevalence and assortment of hemoglobin genotype α-globin gene numbers and G6PD genotypes.

ALPHA GLOBIN	G6PD GENOTYPE			
GENES	A	A-	B	TOTAL
2	9	2	23	34
	27%	6%	68%	10%
3	50	16	94	160
	31%	10%	59%	46%
4	39	23	94	156
	25%	15%	60%	45%
TOTAL	98	41	211	350
	28%	12%	60%	

### The level of Hb S is lower in females than males

**Fig 1:**
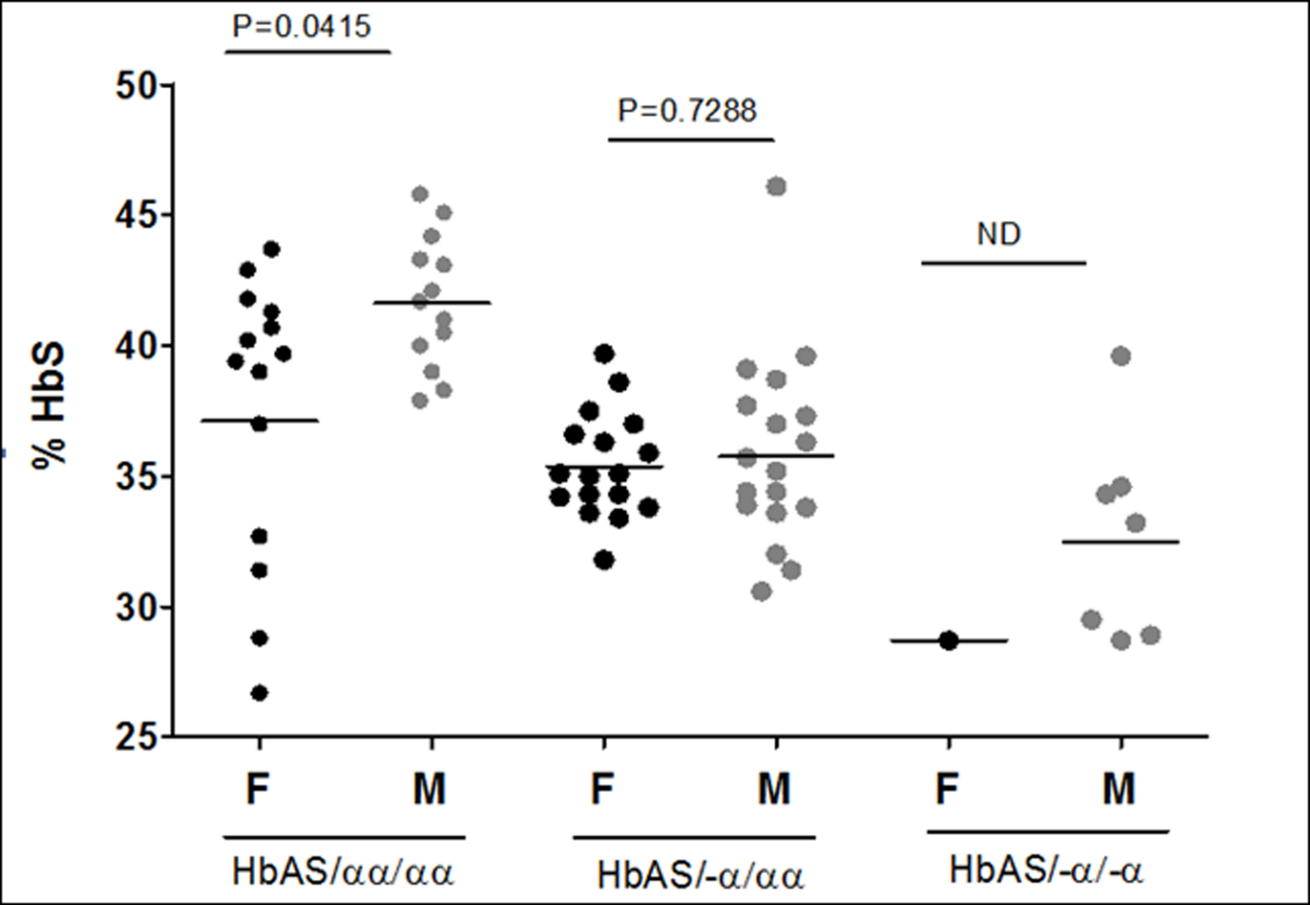
Scatter plots showing the mean proportions of Hb S in females (F) and males (M). In absence of α^+^-thal deletions, gender modified expression of Hb S. More females of HbAS/αα/αα genotype had low Hb S than males and had mean % Hb S of 37.5% ± 5.4 SD, compared to 42.0% ± 2.5 SD in males of same genotype (P = 0.018). For both gender, a loss of one α^+^-thal gene reduced the % Hb S to the same level (mean of 35.4% + x SD for girls and 35.9% x SD for boys). A second loss of α^+^-thal gene reduced the % Hb S even further.

This data came from 70 AS subjects, 32 females and 38 males HPLC BioRad variant which separates and quantifies components of the hemoglobin was therefore used to quantify the proportions of HbS (expressed as a percentage) in the study population. Like in previous studies, the overall proportion of Hb S decreased with the number of alpha globin genes. We used one way ANOVA test to compare the % Hb S between the genders in the three different genotype combinations. More females of HbAS/αα/αα genotype had low Hb S than males (Fig 1) and had mean % Hb S of 37.5% ± 5.4 SD, compared to 42.0% ± 2.5 SD in males of same genotype (P = 0.018). In both gender, these values were reduced further when α^+^-thal genes were deleted (Fig 1). Interestingly, the loss of one α^+^-thal gene reduced the mean Hb S level to about the same level in both gender. For the females, the mean dropped from 37.5% to 35.4% ± y SD and from 42.0% to 35.9% ± y SD for boys. A second loss of α^+^-thal gene reduced the % Hb S even further. Because there was only one female compared to seven males with two α^+^-thal deletions, a fair comparison of % Hb S for the two genders could not be made. The G6PD genotypes did not influence the level of Hb S (data not shown).

### Malaria parasite load of HbAS subjects varies by gender

**Fig 2:**
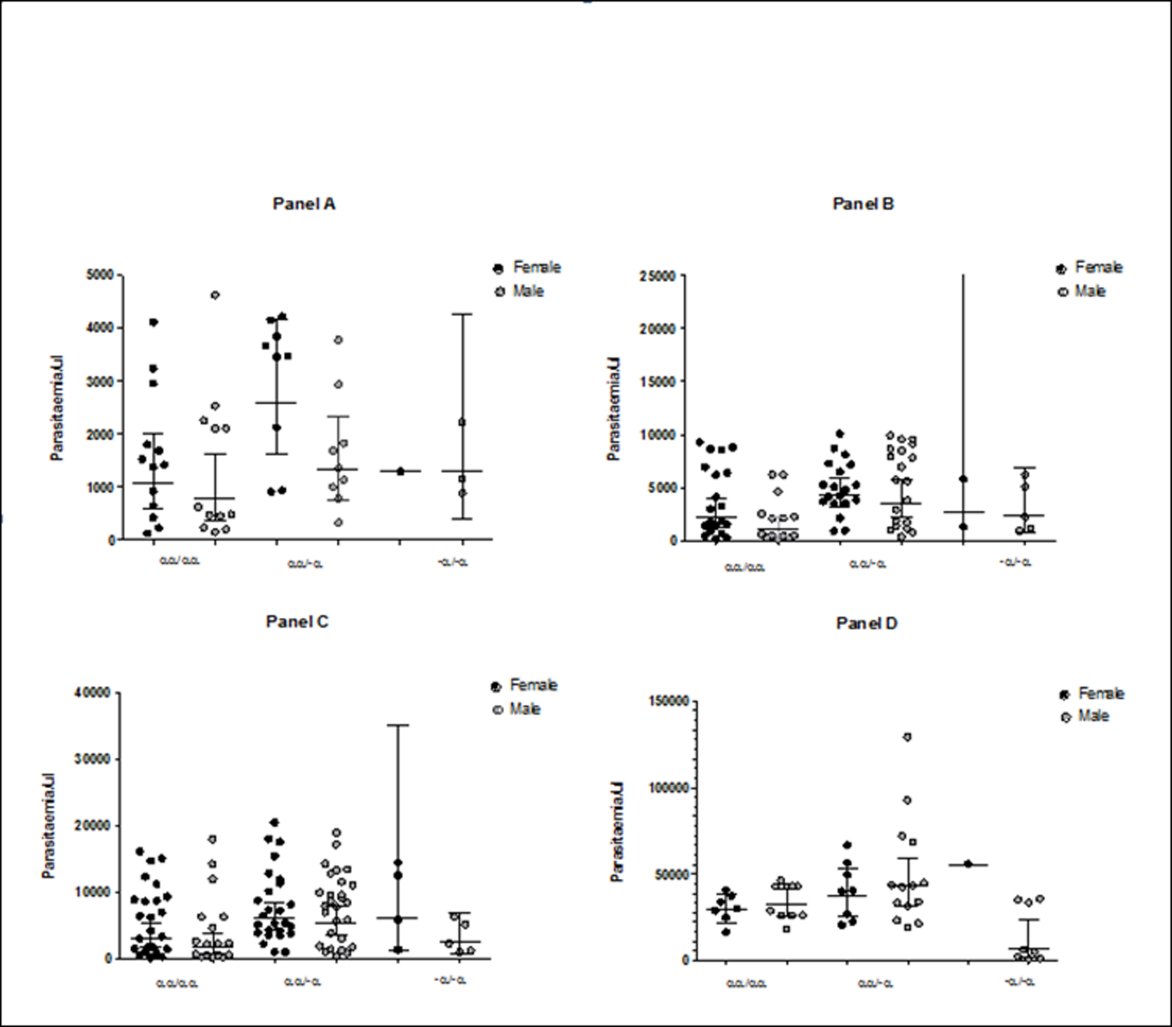
Dot plot showing geometric mean parasite densities within the 25%, 50%, 75% and >75% quartile ranges for boys and girls of HbAS genotype. This data came from multiple sick visits with the 265 days follow-up period. Due to the high variability and with a wide range of parasitemia data, analysis was done at different distribution quartiles. At all the quartile ranges 25%, 50% and 75% (panels A, B and C respectively) the parasite geometric mean for females of HbAS/αα/αα genotype was much higher than for males. As shown in panel C, the median parasite density within the third quartile was two times higher in females of HbAS/αα/αα genotype (3,118, 95% CI: 1779–5465) than for males (1,676, 95% CI: I752-3733). This trend was maintained across the ordered α-thalassemia genotypes (Kurskal-Wallis; 0.0422). A second loss of α^+^-thal gene increased the parasite load even further. Each dot represents parasitemia from a sick visit from the patients.

Multiple malaria parasitaemia data were collected from the 68 of the 70 HbAS study participants who had non-zero malaria parasitemia during sick visits that occurred throughout the 265 days of follow-up. Parasitemia data from every sick visit was included in the analysis. Because malaria parasitemia data tends to be very variable and with a wide range, we looked at parasite density dispersion within the quartiles (25%, 50%, 75% and above 75%). At all the quartile ranges (Fig 2, panels A, B, C and D) the parasite geometric mean for females of HbAS/αα/αα genotype was higher than for males. As shown in Fig 2 panel C, the median parasite density within third quartile was twice as high in females of HbAS/ αα/αα genotype (3,118, 95% CI: 1779–5465) than in males (1,676, 95% CI: I752-3733), though not statistically significant. This trend was maintained across the ordered α-thalassemia genotypes (Kurskal-Wallis; 0.0422). Similar to what was observed with Hb S, i.e the loss of one α^+^-thal gene increased the concentration of Hb S to a similar amount in males and females (Fig 1), the loss of one α^+^-thal gene increased parasite load to about the same geometric mean (5,969 parasites/μL 95% CI: 4309–8270 for females and 5,259 parasites/μL, 95% CI: 3470–7971 for males), thus providing further credence to the possibility that, in this case, the parasite load is dictated by the concentration of Hb S. Above the 75% quartile range, the parasite density between males and females was not different (Fig 2, panel D).

## Discussion

The high prevalence of RBC polymorphisms detected in this study (Tables 1–3): 20% for HbAS, 12% for G6PD*A-, and an even higher prevalence of 3.7 α^+^-thal deletions in heterozygous (-α/αα), 46%) or homozygous (-α/-α, 10%) forms are indicative of the strong selective pressure that malaria has exerted on these traits and are consistent with studies done in other malaria endemic regions in Sub-Saharan Africa [30–34]. Our study found no evidence of departure from independence inheritance of these traits, meaning that particular combinations of traits were not selected for.

Many studies have established the “good” and the “bad” of Hb S. Good because it protects against malaria in individuals of HbAS genotype [3–6]. Bad in that, in situations of reduced oxygen tension in individuals of HbSS genotype, the deoxygenated Hb S polymerizes resulting in rigid distorted red blood cells that can trigger the vaso-occlusive pathologies associated with the sickling phenomenon [35]. That, the intra-erythrocytic concentration of Hb S matters in the protection against malaria and in the degree of sickling is corroborated by a number of studies. For example, individuals who co-inherit HbAS or HbSS and *α*^+^-thal have decreased intra-erythrocytic concentration of Hb S [11, 32] which manifests as loss of protection against malaria in individuals of HbAS genotype [14] or ameriolation of the sickling phenomenon [36, 37] in individuals of HbSS genotype. Also, transgenic mice expressing Hb S were protected against lethal *P*. *yoelii* malaria and the level of protection was proportional to amount of expressed Hb S [16].

As far as we are aware, this is the first report on gender difference in the concentration of Hb S. We think that the reduced Hb S, and thus decreased polymerization, may explain why females have been reported to experience reduced sickle cell crisis compared to males [20, 24, 38–40]. As shown in Fig 1, many more females of HbAS/αα/αα genotype had % Hb S below the group mean compared to males of the same genotype and had mean % Hb S significantly lower than for males. We propose that gender be considered as a genetic modifier of Hb S expression, similar to α^+^-thal. In our study, the resultant deficiency of α-globin chains due to coinheritance of HbAS and α^+^-thal magnified the reduction of Hb S in both genders (Fig 1) and the loss was proportional to the number of α^+^-thal genes lost. Interestingly, the loss of one α^+^-thal gene reduced Hb S to about 35% in both females and males. It is not clear why the reduction was fixed to 35%, but it could probably be due to the manner in which the hemoglobin tetramers combine following loss of a single alpha globin gene [41]. This may be dependent on a threshold concentration of β^A^ and β^S^ rather than on the initial quantity per se.

It is not clear why males would have a higher proportion of Hb S compared to females. One probable explanation is due to the fact that males generally have higher hemoglobin levels than females which has been attributed to hormonal difference [42]. However, a previous study by Hawkins et al (1954) showed that the gender difference in hemoglobin levels does not show until the age of 12 years [43]. Our study population involved boys and girls under the age of 4 (12–47 months) at which age, the hemoglobin levels are similar. Other explanations that we have considered include increased expression of microRNAs [44] probably attributable to hormones. In a study reported by LaMonte, expression level of sickle cell erythrocyte microRNAs was higher in individuals of HbSS genotype compared to HbAS and was lowest in the HbAA genotype [45]. It is therefore not inconceivable that males over express β^S^ globin gene that would lead to higher levels of Hb S compared to females. But at such an early age when hormonal differences between boys and girls are not obvious, it is unclear what would cause higher levels of Hb S in boys compared to girls.

Although the vaccine study under which the participants who contributed these data did not show differences in malaria parasite load between the randomized study arms [26], we were interested in evaluating the effect of the reduction in % Hb S on parasite densities in males and females. Because malaria parasitemia determination tend to be very variable, we analyzed the parasite load over the different quartiles ranges. As shown in Fig 2 and mirroring the reduced Hb S in females, we found a commensurate possible loss of protection against malaria in the females. This is in line with a study by Hood *et al*. that showed that transgenic mice expressing Hb S were protected against lethal *P*. *yoelii* malaria and the level of protection was proportional to amount of expressed Hb S [16]. Deletion of α-globin genes led to further reduction of Hb S in both gender and a concomitant further loss of protection against malaria. Similar to the situation observed with Hb S level after the loss of one α^+^-thal gene (reduction to 35% irrespective of initial level), the parasitemia load in females and males with one α^+^-thal deletion was about the same (~5,000 parasites per μL). Clearly, the amount of Hb S seems to dictate the parasitemia level.

One of the limitations of this study is the small sample sizes for the numbers of subjects and visits for the combinations of HbAS genotype and homozygous α^+^-thal. This complicated the analysis of interactions among the predictors.

### Conclusions

In conclusion, for the first time, this study reports on gender difference in the intra-erythrocytic concentration of Hb S, lower in females than males. Taken together, our data indicate that gender is a genetic modifier of Hb S expression, similar to α^+^-thal gene deletion. We think that the gender difference in concentration of Hb S explains why females have reduced sickle cell crisis compared to males.

## References

[1] Weatherall DJ. Genetic variation and susceptibility to infection: the red cell and malaria. British journal of haematology. 2008;141(3):276–86. Epub 2008/04/16. 10.1111/j.1365-2141.2008.07085.x . 18410566

[2] Hedrick PW. Population genetics of malaria resistance in humans. Heredity (Edinb). 2011;107(4):283–304. 10.1038/hdy.2011.16 ; PubMed Central PMCID: PMCPMC3182497. 21427751 PMC3182497

[3] Allison AC. Protection afforded by sickle-cell trait against subtertian malareal infection. Br Med J. 1954;1(4857):290–4. ; PubMed Central PMCID: PMCPMC2093356. 13115700 10.1136/bmj.1.4857.290 PMC2093356

[4] Ackerman H, Usen S, Jallow M, Sisay-Joof F, Pinder M, Kwiatkowski DP. A comparison of case-control and family-based association methods: the example of sickle-cell and malaria. Ann Hum Genet. 2005;69(Pt 5):559–65. 10.1111/j.1529-8817.2005.00180.x . 16138914

[5] Aidoo M, Terlouw DJ, Kolczak MS, McElroy PD, ter Kuile FO, Kariuki S, et al Protective effects of the sickle cell gene against malaria morbidity and mortality. Lancet. 2002;359(9314):1311–2. 10.1016/S0140-6736(02)08273-9 . 11965279

[6] Willcox M, Bjorkman A, Brohult J, Pehrson PO, Rombo L, Bengtsson E. A case-control study in northern Liberia of Plasmodium falciparum malaria in haemoglobin S and beta-thalassaemia traits. Ann Trop Med Parasitol. 1983;77(3):239–46. . 6354114 10.1080/00034983.1983.11811704

[7] Guindo A. X-Linked G6PD Deficiency Protects Hemizygous Males but Not Heterozygous Females against Severe Malaria. 2007;4(3). 10.1371/journal.pmed.0040066 ; PubMed Central PMCID: PMCPmc1820604. 17355169 PMC1820604

[8] Uyoga S, Ndila CM, Macharia AW, Nyutu G, Shah S, Peshu N, et al Glucose-6-phosphate dehydrogenase deficiency and the risk of malaria and other diseases in children in Kenya: a case-control and a cohort study. The Lancet Haematology. 2015;2(10):e437–44. Epub 2015/12/22. 10.1016/S2352-3026(15)00152-0 ; PubMed Central PMCID: PMCPMC4703047. 26686045 PMC4703047

[9] Ruwende C, Khoo SC, Snow RW, Yates SN, Kwiatkowski D, Gupta S, et al Natural selection of hemi- and heterozygotes for G6PD deficiency in Africa by resistance to severe malaria. Nature. 1995;376(6537):246–9. Epub 1995/07/20. 10.1038/376246a0 . 7617034

[10] Brittenham G, Lozoff B, Harris JW, Mayson SM, Miller A, Huisman TH. Sickle cell anemia and trait in southern India: further studies. American journal of hematology. 1979;6(2):107–23. Epub 1979/01/01. . 474571 10.1002/ajh.2830060203

[11] Higgs DR, Aldridge BE, Lamb J, Clegg JB, Weatherall DJ, Hayes RJ, et al The interaction of alpha-thalassemia and homozygous sickle-cell disease. The New England journal of medicine. 1982;306(24):1441–6. Epub 1982/06/17. 10.1056/NEJM198206173062402 . 6176865

[12] Mouele R, Pambou O, Feingold J, Galacteros F. alpha-thalassemia in Bantu population from Congo-Brazzaville: its interaction with sickle cell anemia. Hum Hered. 2000;50(2):118–25. Epub 2000/05/09. 10.1159/000022899 . 10799970

[13] Wambua S, Mwacharo J, Uyoga S, Macharia A, Williams TN. Co-inheritance of alpha+-thalassaemia and sickle trait results in specific effects on haematological parameters. Br J Haematol. 2006;133(2):206–9. Epub 2006/04/14. 10.1111/j.1365-2141.2006.06006.x ; PubMed Central PMCID: PMCPmc4394356. 16611313 PMC4394356

[14] Williams TN, Mwangi TW, Wambua S, Peto TE, Weatherall DJ, Gupta S, et al Negative epistasis between the malaria-protective effects of alpha+-thalassemia and the sickle cell trait. Nature genetics. 2005;37(11):1253–7. Epub 2005/10/18. 10.1038/ng1660 ; PubMed Central PMCID: PMCPMC3521056. 16227994 PMC3521056

[15] Lopera-Mesa TM, Doumbia S, Konate D, Anderson JM, Doumbouya M, Keita AS, et al Effect of red blood cell variants on childhood malaria in Mali: a prospective cohort study. The Lancet Haematology. 2015;2(4):e140–9. Epub 2015/12/22. 10.1016/S2352-3026(15)00043-5 ; PubMed Central PMCID: PMCPMC4418020. 26687956 PMC4418020

[16] Hood AT, Fabry ME, Costantini F, Nagel RL, Shear HL. Protection from lethal malaria in transgenic mice expressing sickle hemoglobin. Blood. 1996;87(4):1600–3. . 8608253

[17] Baum KF, Dunn DT, Maude GH, Serjeant GR. The painful crisis of homozygous sickle cell disease: A study of risk factors. Archives of Internal Medicine. 1987;147(7):1231–4. 10.1001/archinte.1987.00370070045007 3606281

[18] Platt OS, Brambilla DJ, Rosse WF, Milner PF, Castro O, Steinberg MH, et al Mortality In Sickle Cell Disease—Life Expectancy and Risk Factors for Early Death. New England Journal of Medicine. 1994;330(23):1639–44. 10.1056/NEJM199406093302303 . 7993409

[19] Platt OS, Thorington BD, Brambilla DJ, Milner PF, Rosse WF, Vichinsky E, et al Pain in Sickle Cell Disease. New England Journal of Medicine. 1991;325(1):11–6. 10.1056/NEJM199107043250103 . 1710777

[20] Kamble M, Chatruvedi P. Epidemiology of sickle cell disease in a rural hospital of central India. Indian pediatrics. 2000;37(4):391–6. Epub 2000/04/26. . 10781232

[21] Chang YC, Smith KD, Moore RD, Serjeant GR, Dover GJ. An analysis of fetal hemoglobin variation in sickle cell disease: the relative contributions of the X-linked factor, beta-globin haplotypes, alpha-globin gene number, gender, and age. Blood. 1995;85(4):1111–7. Epub 1995/02/15. . 7531513

[22] Dover GJ, Smith KD, Chang YC, Purvis S, Mays A, Meyers DA, et al Fetal hemoglobin levels in sickle cell disease and normal individuals are partially controlled by an X-linked gene located at Xp22.2. Blood. 1992;80(3):816–24. Epub 1992/08/01. . 1379090

[23] Steinberg MH, Hsu H, Nagel RL, Milner PF, Adams JG, Benjamin L, et al Gender and haplotype effects upon hematological manifestations of adult sickle cell anemia. American journal of hematology. 1995;48(3):175–81. Epub 1995/03/01. . 7532353 10.1002/ajh.2830480307

[24] Gladwin MT, Schechter AN, Ognibene FP, Coles WA, Reiter CD, Schenke WH, et al Divergent nitric oxide bioavailability in men and women with sickle cell disease. Circulation. 2003;107(2):271–8. Epub 2003/01/23. . 12538427 10.1161/01.cir.0000044943.12533.a8

[25] Vekilov PG. Sickle-cell haemoglobin polymerization: is it the primary pathogenic event of sickle-cell anaemia? Br J Haematol. 2007;139(2):173–84. 10.1111/j.1365-2141.2007.06794.x . 17897293

[26] Ogutu BR, Apollo OJ, McKinney D, Okoth W, Siangla J, Dubovsky F, et al Blood stage malaria vaccine eliciting high antigen-specific antibody concentrations confers no protection to young children in Western Kenya. PloS one. 2009;4(3):e4708 Epub 2009/03/06. 10.1371/journal.pone.0004708 ; PubMed Central PMCID: PMCPMC2650803. 19262754 PMC2650803

[27] Guindo A, Fairhurst RM, Doumbo OK, Wellems TE, Diallo DA. X-linked G6PD deficiency protects hemizygous males but not heterozygous females against severe malaria. PLoS Med. 2007;4(3):e66 10.1371/journal.pmed.0040066 ; PubMed Central PMCID: PMCPMC1820604. 17355169 PMC1820604

[28] Ohrt C, Obare P, Nanakorn A, Adhiambo C, Awuondo K, O'Meara WP, et al Establishing a malaria diagnostics centre of excellence in Kisumu, Kenya. Malar J. 2007;6:79 Epub 2007/06/15. 10.1186/1475-2875-6-79 ; PubMed Central PMCID: PMCPmc1933544. 17565676 PMC1933544

[29] Ohrt C, O'Meara WP, Remich S, McEvoy P, Ogutu B, Mtalib R, et al Pilot assessment of the sensitivity of the malaria thin film. Malar J. 2008;7:22 Epub 2008/01/30. 10.1186/1475-2875-7-22 ; PubMed Central PMCID: PMCPmc2266769. 18226243 PMC2266769

[30] Waitumbi JN, Kifude CM, Withers MR, Polhemus ME, Heppner DG Jr., Ogutu BR. Hb G-Philadelphia or Stanleyville II? When the phenotype and genotype do not agree. European journal of haematology. 2007;79(2):177–8. Epub 2007/07/05. 10.1111/j.1600-0609.2007.00879.x . 17608715

[31] Carter N, Pamba A, Duparc S, Waitumbi JN. Frequency of glucose-6-phosphate dehydrogenase deficiency in malaria patients from six African countries enrolled in two randomized anti-malarial clinical trials. Malaria journal. 2011;10:241 Epub 2011/08/19. 10.1186/1475-2875-10-241 ; PubMed Central PMCID: PMCPMC3188486. 21849081 PMC3188486

[32] Embury SH, Dozy AM, Miller J, Davis JR Jr., Kleman KM, Preisler H, et al Concurrent sickle-cell anemia and alpha-thalassemia: effect on severity of anemia. The New England journal of medicine. 1982;306(5):270–4. Epub 1982/02/04. 10.1056/NEJM198202043060504 . 6172710

[33] Williams TN, Mwangi TW, Wambua S, Alexander ND, Kortok M, Snow RW, et al Sickle cell trait and the risk of Plasmodium falciparum malaria and other childhood diseases. The Journal of infectious diseases. 2005;192(1):178–86. Epub 2005/06/09. 10.1086/430744 ; PubMed Central PMCID: PMCPMC3545189. 15942909 PMC3545189

[34] Millimono TS, Loua KM, Rath SL, Relvas L, Bento C, Diakite M, et al High prevalence of hemoglobin disorders and glucose-6-phosphate dehydrogenase (G6PD) deficiency in the Republic of Guinea (West Africa). Hemoglobin. 2012;36(1):25–37. Epub 2011/09/21. 10.3109/03630269.2011.600491 . 21929367

[35] Bunn HF. Pathogenesis and treatment of sickle cell disease. The New England journal of medicine. 1997;337(11):762–9. Epub 1997/09/11. 10.1056/NEJM199709113371107 . 9287233

[36] Sheehan VA, Luo Z, Flanagan JM, Howard TA, Thompson BW, Wang WC, et al Genetic modifiers of sickle cell anemia in the BABY HUG cohort: influence on laboratory and clinical phenotypes. American journal of hematology. 2013;88(7):571–6. Epub 2013/04/23. 10.1002/ajh.23457 . 23606168

[37] Embury SH. Age-dependent changes in the membrane surface area: sickle red blood cell volume may account for differential clinical effects of coinherited alpha thalassemia on sickle cell anemia. European journal of haematology. 2012;88(4):363–4. Epub 2011/12/16. 10.1111/j.1600-0609.2011.01743.x . 22168478

[38] Baum KF, Dunn DT, Maude GH, Serjeant GR. The painful crisis of homozygous sickle cell disease. A study of the risk factors. Arch Intern Med. 1987;147(7):1231–4. Epub 1987/07/01. . 3606281

[39] Platt OS, Brambilla DJ, Rosse WF, Milner PF, Castro O, Steinberg MH, et al Mortality in sickle cell disease. Life expectancy and risk factors for early death. The New England journal of medicine. 1994;330(23):1639–44. Epub 1994/06/09. 10.1056/NEJM199406093302303 . 7993409

[40] Garvin J. Gender-Specific Aspects of Pediatric Hematology and Oncology. 2nd ed In Legato MJ (Ed.) PoG-SM, editor: Elsevier: Academic Press; 2010. 52–3 p.

[41] Bunn HF, McDonald MJ. Electrostatic interactions in the assembly of haemoglobin. Nature. 1983;306(5942):498–500. Epub 1983/12/01. . 6646230 10.1038/306498a0

[42] Murphy WG. The sex difference in haemoglobin levels in adults—mechanisms, causes, and consequences. Blood reviews. 2014;28(2):41–7. Epub 2014/02/05. 10.1016/j.blre.2013.12.003 . 24491804

[43] Hawkins WW, Speck E, Leonard VG. Variation of the hemoglobin level with age and sex. Blood. 1954;9(10):999–1007. Epub 1954/10/01. . 13208753

[44] Sharma S, Eghbali M. Influence of sex differences on microRNA gene regulation in disease. Biol Sex Differ. 2014;5(1):3 10.1186/2042-6410-5-3 ; PubMed Central PMCID: PMCPMC3912347. 24484532 PMC3912347

[45] LaMonte G, Philip N, Reardon J, Lacsina JR, Majoros W, Chapman L, et al Translocation of sickle cell erythrocyte microRNAs into Plasmodium falciparum inhibits parasite translation and contributes to malaria resistance. Cell Host Microbe. 2012;12(2):187–99. 10.1016/j.chom.2012.06.007 ; PubMed Central PMCID: PMCPMC3442262. 22901539 PMC3442262

